# Valuing an urban marathon as a public sport service: evidence from the Seoul international marathon

**DOI:** 10.3389/fspor.2026.1896016

**Published:** 2026-09-30

**Authors:** Sang-hyun Shin, Jae-eun Shin, Timothy Baghurst

**Affiliations:** 1 Chamirisa College of Liberal Arts, Duk-sung Women's University, Seoul, Republic of Korea; 2 College of Sport Science, Chung-Ang University, Seoul, Republic of Korea; 3 College of Education, Health, and Human Sciences, Florida State University, Tallahassee, FL, United States

**Keywords:** contingent valuation method (CVM), focus group interviews (FGI), non-market economic valuation, public sport services, urban sport infrastructure

## Abstract

This study examined the nonmarket value of the Seoul International Marathon (SIM) as part of the city's sport infrastructure and public sport service provision, using the Contingent Valuation Method (CVM) to estimate Seoul residents' willingness to pay for the event. An online survey of 1,250 adult residents employed a double-bounded dichotomous-choice format in a hypothetical tax-based donation scenario. A logit model was used to explore which factors shape willingness to pay, and bid level, interest in marathon events, satisfaction with related infrastructure, event perception, occupation, and gender emerged as significant predictors. The estimated individual contribution ranged from KRW 15,781.51 (USD 11.34), the median WTP at KRW 14,394.95 (USD 10.35), and the truncated WTP at KRW 4,747.86 (USD 3.41). The findings indicate that the SIM delivers substantial nonmarket benefits for the host city and that residents' positive views of the event and its supporting environment are closely linked to their readiness to contribute. These results underline the potential of large-scale road races to strengthen urban sport infrastructure and provide practical guidance for sport managers and local governments when deciding on public funding, route and facility improvements, event design, and the development of citywide running programmes. The study also shows how resident-based valuation can be incorporated into planning processes to align sport event strategies with broader urban sport and health objectives. Preprint statement: An earlier version of this manuscript was posted as a preprint on Research Square (DOI: 10.21203/rs.3.rs-8746817/v1).

## Introduction

Public sports services represent government-supported initiatives that promote public health, strengthen community identity, and enhance opportunities for leisure participation (1, 2). These services extend beyond the physical provision of sport facilities and function as policy instruments that generate intangible social value, such as community cohesion, civic pride, and the development of a healthy civil society (3–5). From a policy studies perspective, public sports services operate within a governance structure that integrates principles of new public management with collaboration among multiple actors. This requires a systematic evaluation of both their economic and social contributions.

Large-scale urban sports events hosted by municipal governments exhibit these characteristics and contribute to enhanced access to physical activity, tourism revitalization, increased economic activity, and a strengthened sense of community identity. Because these events are nonmarket goods, their economic value cannot be observed through conventional market transactions. Therefore, nonmarket valuation approaches are necessary to determine their broader societal effects, support evidence-based decision-making, and reduce inefficiencies in public resource allocation (3, 6–8).

The Seoul International Marathon (SIM) offers a compelling context for such evaluation. Established in 1931, the event is internationally recognized as one of the oldest full-course marathons. It is registered alongside the Boston Marathon and the Athens Marathon as one of the three major cultural heritage marathon events (9). Its long history, cultural significance, and contribution to civic identity position it as an important public service asset.

Recent growth in global participation in running has also intensified interest in understanding the broader economic effects of marathon events, including their contributions to tourism, local business growth, and the international visibility of host cities (10, 11). Research evaluating the economic contributions of major world marathons has demonstrated substantial impacts, with events such as the New York City Marathon generating hundreds of millions of dollars in economic activity and contributing significantly to job creation and place branding (7, 12). Therefore, these trends underscore the importance of objective assessment tools in evaluating public value and informing resource planning for large-scale sporting events.

Against this backdrop of growing academic interest and increasing policy demand, evaluating the economic value of the SIM using the Contingent Valuation Method (CVM) is essential for providing scientific evidence of its social and economic value and for supporting evidence-based policymaking and event planning. Therefore, the purpose of this study was to estimate the non-market value of the SIM as a public sport service by applying the CVM, a widely accepted approach for valuing non-market goods and services. Furthermore, this study sought to provide practical implications for policymakers and event managers while offering an empirical foundation for the development of future public sport services and evidence-informed sport policy.

## Literature review

### Valuation of nonmarket goods and the contingent valuation method

The Contingent Valuation Method (CVM) provides a well-established framework for valuing nonmarket goods and is widely used to estimate both use and non-use benefits (13, 14). Originating from early environmental economics studies (15, 16), CVM gained prominence following the Exxon Valdez oil spill, when a United States federal review committee concluded that, when properly applied, CVM is theoretically valid for estimating compensation for nonmarket losses (17). CVM constructs a hypothetical market through survey-based elicitation of willingness to pay, using econometric models to estimate compensating variation and equivalent variation (17, 18). Its flexibility has supported applications across environmental resources, cultural assets, public services, and major sports events (19–21).

Nevertheless, CVM requires careful survey design because it relies on stated preferences. Methodological challenges include strategic bias, design bias, and hypothetical market bias (17, 22). To address these issues, contemporary best practices emphasize the use of dichotomous choice formats, realistic scenario construction, and preliminary testing of bid amounts. The dichotomous choice method, particularly in its double-bounded form, has been shown to reduce cognitive burden, increase realism, and improve statistical efficiency (23–25).

### CVM applications in sport facilities, mega-events and elite sport programmes

In the sport context, CVM has been widely used to measure the economic value of sport facilities, mega-events, and elite sport programmes. Johnson and Whitehead (26), for example, applied CVM to estimate the public good value of professional sport stadiums and showed that residents derive significant nonmarket benefits from their existence. Atkinson et al. (19) used CVM to assess the intangible benefits associated with London's bid for the 2012 Olympic Games, identifying positive annual willingness to pay (WTP) among residents in several British cities. Humphreys et al. (21) analysed Canadian residents' WTP for the “Own the Podium” programme associated with the Vancouver 2010 Winter Olympics. They reported that average WTP increased substantially after the Games, reflecting perceived benefits from improved Olympic performance. In the context of mega-sport events, Süssmuth et al. (27) estimated German residents' willingness to pay (WTP) for hosting the 2006 FIFA World Cup. Before the tournament, the total WTP for a successful, feel-good event was approximately $467 million. In contrast, a follow-up survey conducted immediately after the event indicated that WTP had risen to about $1.1 billion, highlighting the substantial nonmarket benefits associated with hosting mega-events. Building on this line of research, Kwon and Shin (28) employed CVM to estimate Korean citizens' WTP for elite athlete development. They compared it with actual government expenditure, finding that the perceived social benefits of national team development were approximately 1.4 times higher than public investment in the sports they supported. In the Korean context, CVM has also been used to evaluate the ecotourism use of Olympic infrastructure, the feasibility of motorsport events, and the nonmarket value of various sport-related policy initiatives (29, 30).

Given these theoretical and policy considerations, the purpose of the present study was to apply the CVM to estimate the nonmarket economic value of the SIM and to identify the socio-economic and perceptual factors that shape willingness to pay (WTP) for the event. By constructing a hypothetical market with a rigorously designed scenario and employing a double-bounded dichotomous choice format, supported by a preliminary bid-testing process, we aimed to produce scientifically grounded estimates to inform public funding decisions, long-term event planning, infrastructure development, and the strategic management of urban sport services. This integrated approach provides empirical evidence that supports rational decision-making and enhances the policy relevance of major urban sports events.

## Method

### Research design

This study employed a quantitative cross-sectional survey design using the contingent valuation method (CVM) to estimate citizens' willingness to pay (WTP) for the Seoul International Marathon (SIM) as a public sport service. A cross-sectional survey design is appropriate for examining individuals' perceptions and stated preferences at a specific point in time and is widely used in quantitative social science research (31). The study employed a double-bounded dichotomous-choice elicitation format to reduce respondents' cognitive burden, approximate real-world payment decisions, and improve statistical efficiency relative to open-ended or single-bounded formats (23, 24). A logit model estimated by maximum likelihood was used to analyse the binary WTP responses, consistent with standard practice in CVM applications where the probability of accepting a given bid is modelled as a function of bid level and respondent characteristics (22).

### Instruments and procedure

The economic value of the SIM was assessed using the contingent valuation method (CVM). Before conducting the analysis, ethical approval was obtained from the Institutional Review Board of Duksung Women's University (IRB No. 2025-012-029-B). stages: (1) constructing a scenario to establish a hypothetical market, (2) developing a questionnaire. Data collection took place over one month, beginning on June 1, 2025. During this period, written consent and self-administered questionnaires were collected simultaneously, both online and directly, from all study participants. The analytical procedure comprised four the questionnaire, (3) designing the sampling strategy and administering the survey, (4) specifying and estimating the willingness-to-pay (WTP) function. The overall analytical process is illustrated in Figure 1.

**Figure 1:**
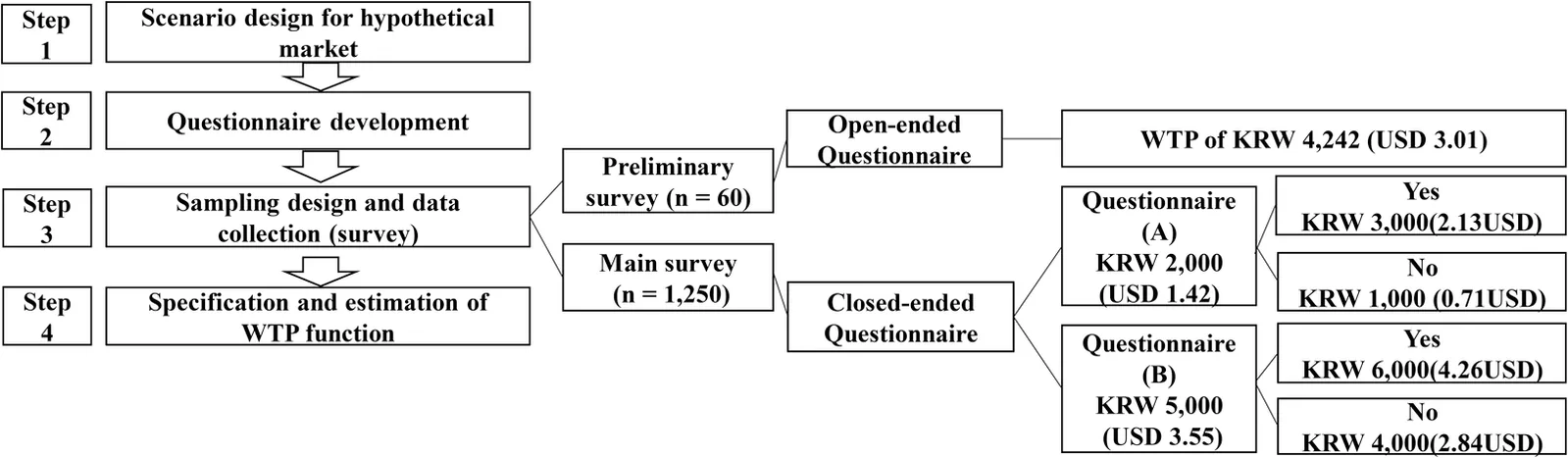
Overall procedure.

### Participants

One of the most important considerations in contingent valuation studies is the selection of the population, as it influences both the sampling unit and the sample size. In this study, although the SIM is an international-scale event, the population was defined as citizens aged 18 years or older residing in Seoul's 25 districts, as the event is hosted within the city. Before beginning the survey, the researcher provided all participants with a consent form and explained in detail the study's purpose, privacy protections, and how the results would be used. The sample's general demographic characteristics are presented in Table 1.

**Table 1:** Demographic characteristics of respondents.

Variable	Category	Frequency (n)	Percentage (%)	Variable	Category	Frequency (n)	Percentage (%)	Variable	Category	Frequency (n)	Percentage (%)
Questionnaire	A	625	50.0	Occupation	Unemployed	18	3.4	Interest in Health	Very interested	769	61.5
	B	625	50.0		Office/Administrative	16	3.1		Interested	269	21.5
Willingness to Pay	Yes	829	66.3		Housewife	291	55.7		Neutral	152	12.2
	No	421	33.7		University student	76	14.6		Not interested	50	4.0
Gender	Male	530	42.4		Self-employed	79	15.1		Not interested at all	10	0.8
	Female	720	57.6		Professional	22	4.2	Interest in Marathon	Very interested	415	33.2
Age	20s	153	12.2		Research/Technical	11	2.1		Interested	284	22.7
	30s	210	16.8		Sales/Service	7	1.3		Neutral	405	32.4
	40s	396	31.7		Manufacturing Technician	2	0.4		Not interested	129	10.3
	50s	391	31.3	Monthly Income	Less than KRW 1 million	229	18.3		Not interested at all	17	1.4
	60s	100	8.0		KRW 1–2 million	296	23.7	Past Exercise Prescription	Yes	131	25.1
Education	No formal education	18	1.4		KRW 2–3 million	234	18.7		No	391	74.9
	Elementary school graduate	88	7.0		KRW 3–4 million	248	19.8	Experience Membership in a Sports Club	Yes	265	50.8
	Middle school graduate	106	8.5		KRW 4–5 million	72	5.8		No	257	49.2
	High school graduate	686	54.9		KRW 5–6 million	70	5.6	Intention to Reparticipate	Strong intention	377	72.2
	Currently enrolled in university	79	6.3		KRW 6–7 million	13	1.1		Willing to participate	118	22.6
	University graduate	261	20.9		More than KRW 7 million	88	7.0		Neutral	18	3.4
	Currently enrolled in graduate school	6	0.5	Perceived Necessity of National Fitness 100	Necessary	396	75.9		Not willing	9	1.7
	Graduate school graduate	6	0.5		Not necessary	126	24.1	Intention to Recommend	Strong intention	322	61.7
				Participation Frequency	1 time	333	63.8		Willing to recommend	142	27.2
					2 times	138	26.4				
					3 times	51	9.8		Neutral	42	8.0
									Not willing	16	3.1

Percentages for the relevant subgroups are based on 522 respondents.

### Scenario setting for WTP

The SIM was selected as the subject of this study, and a hypothetical market was constructed to estimate its non-market economic value as a public sport service. To elicit citizens' willingness to pay (WTP), the qualitative improvement of the event and its supporting infrastructure was presented as the contingent commodity in a tax-based donation scenario. In line with CVM best practices, particular attention was paid to survey design, as the choice of elicitation format and the realism of the scenario have a substantial impact on the validity and reliability of the results (17, 32). Accordingly, the questionnaire was developed based on the theoretical framework of CVM and previous studies on the valuation of public goods and sport events, following the principles proposed by Carson (13) and the recommendations of the NOAA Panel (33), which emphasize understandable, valid, and meaningful scenarios and the provision of sufficient but not excessive information to respondents.

The survey was structured to provide sufficient but not excessive information about the SIM project, to minimize strategic and design-related biases, and to enable respondents to make informed judgements about their willingness to pay.

To further examine and refine the scenario, two rounds of focus group interviews (FGIs) were conducted between March 25 and April 5, 2024, with members of the SIM Organizing Committee, officials from the Seoul Sports Council, and staff from the Seoul Tourism and Sports Department. Feedback from these FGIs was used to adjust the description of the project, the payment vehicle, and the wording of key questions, ensuring that the hypothetical market conditions were realistic and that respondents could state their WTP under clearly defined assumptions. In addition to WTP questions, items on health interest, marathon participation, and satisfaction with the supporting infrastructure were included to prevent respondents from focusing solely on monetary aspects.

The final survey employed a double-bounded dichotomous-choice elicitation format, asking participants how much they would be willing to pay as an income-tax donation to the SIM project. This approach has been recognized as one of the most effective methods for minimizing various types of bias, as it reduces cognitive burden, resembles real market purchasing situations, and improves statistical efficiency compared with single-bounded formats (23, 25, 34, 35). By presenting bid amounts pretested in a preliminary survey and using a double-bounded design, the study sought to obtain WTP estimates that more closely reflect feasible payment behavior while mitigating starting-point and design-related biases.

### Preliminary survey

The double-bounded dichotomous choice WTP estimation method is subject to starting-point bias, whereby the results vary with the initial amount presented to respondents, making the size of the initial bid highly significant (36, 37). To eliminate starting-point bias, previous studies have generally conducted a preliminary survey using an open-ended format to estimate respondents' WTP before administering the primary survey with dichotomous-choice questions (38).

To determine the initial bid amount, a preliminary survey was conducted with 60 adult Seoul residents recruited via convenience sampling. The survey employed an open-ended question format (see Supplementary Appendix A). The questionnaire included five questions from SIM's perception survey, such as “*What do you think of the Seoul International Marathon*?” and “*If funding were to be raised through donations for the successful hosting of the Seoul International Marathon, would you be willing to pay? How much would you be willing to pay?*”

The responses obtained from the preliminary survey were analyzed using a conventional content analysis approach. First, all responses were reviewed to identify incomplete, inconsistent, or protest responses. Second, valid responses to the open-ended willingness-to-pay question were summarized by examining their frequency distribution and central tendency to determine an appropriate initial bid amount for the main survey. Responses to the open-ended perception questions were independently reviewed by the research team and grouped into common categories based on recurring ideas and expressions. The findings from this process were used to improve the wording, clarity, and realism of the hypothetical market scenario and questionnaire items before the final survey was administered.

The results of the preliminary survey indicated minimum and maximum WTP values of KRW 1,000 (USD 0.71) and KRW 10,000 (USD 6.86), respectively, with an average WTP of KRW 4,242 (USD 3.01). The preliminary survey scenario used in this study is as follows.

### Primary survey questions

Based on preliminary findings, the main survey employed a closed-ended questionnaire in a double-bounded dichotomous choice format (see Appendices B and C). In version A of the questionnaire, the initial bid was set at KRW 2,000 (USD 1.37). Respondents willing to pay this amount were then offered an increased bid of KRW 3,000 (USD 2.06), whereas those unwilling to pay KRW 2,000 were presented with a decreased bid of KRW 1,000 (USD 0.69).

In version B, the initial bid was set at KRW 5,000 (USD 3.43). Respondents who accepted this amount were subsequently offered KRW 6,000 (USD 4.12), while those who declined were offered a lower bid of KRW 4,000 (USD 2.75). Accordingly, each respondent's true WTP was designed to fall into one of the following intervals:

Questionnaire A (Supplementary Appendix B): A < 1,000; 1,000 ≤ A < 2,000; 2,000 ≤ A < 3,000; A ≥ 3,000

Questionnaire B (Supplementary Appendix C): B < 4,000; 4,000 ≤ B < 5,000; 5,000 ≤ B < 6,000; B ≥ 6,000

By assigning version A (Supplementary Appendix B) to relatively lower bid levels (1,000–3,000 KRW) and version B (Supplementary Appendix C) to higher bid levels (4,000–6,000 KRW), the design sought to obtain information on a wider range of the WTP distribution and to reduce the influence of starting point bias through the use of two different initial bids within a double-bounded, multi-bid structure. For convenience, this configuration is referred to as a “double-bid design” in the present study (Table 2).

**Table 2:** WTP Bid amounts.

Preliminary Survey (Open-ended Questionnaire)	Main Survey (Closed-ended Questionnaire)			
	1st Question		2nd Question	
	Initial Bid		Yes	No
1,000≦A≦10,000 WTP = 4,242($3.01)	A	2,000 ($1.42)	3,000 ($2.13)	1,000 ($0.71)
	B	5,000 ($3.55)	6,000 ($4.26)	4,000 ($2.84)

In addition to questions about WTP, participants were asked up to six questions about the intangible benefits of the SIM based on their responses. These included ascertaining whether the participant would be willing to make a financial contribution for the successful hosting of the marathon, how much or little they would be willing to contribute, and the main reason they would be willing/unwilling to pay an income tax contribution.

### Data analysis

A convenience sampling method, a form of non-probability sampling, was employed. The results from 1,250 questionnaires were analyzed using SPSS 26.0. A logit model was used for estimation, incorporating variables whose significance had been previously verified in studies.

#### WTP analysis model and estimation method

In this study, a “Yes” response to the proposed bid indicated that the respondent was willing to pay the amount B. A respondent was assumed to accept the proposed bid when the utility associated with supporting the SIM exceeded the utility associated with rejecting it. This condition can be expressed using the following indirect utility function:

v₁(1, I − B; X) + ε₁ ≥ v₀(0, I; X) + ε₀. (1)

Here, I represents the respondent's income, B represents the proposed bid amount, X is a vector of the respondent's socioeconomic and perceptual characteristics, and ε₁ and ε₀ are random error terms.

The subscripts 1 and 0 represent acceptance and rejection of the proposed bid, respectively.

The difference between the deterministic components of the two utility functions is defined as:

Δv (B, X) = v₁ (1, I − B; X) − v₀(0, I; X).

Defining ε = ε₀ − ε₁, the probability that a respondent accepts the proposed bid can be expressed as:

P(Y = 1 | B, X) = Pr [ε ≤ Δv(B, X)] = Fε [Δv(B, X)]. (2)

Assuming that the difference in the random error terms follows a logistic distribution, the probability of accepting the proposed bid is specified as:

P(Y = 1 | B, X) = Fε(Δv) = 1 / [1 + exp(−Δv)].(3)

The systematic utility difference is specified as:

Δv = α + β_B B + β′X. (4)

Here, α is the intercept, β_B is the coefficient associated with the bid amount, β is the vector of coefficients associated with the explanatory variables, and X is the vector of respondent characteristics listed in Table 3. These characteristics include health interest, marathon interest, infrastructure satisfaction, sports club membership, event participation, perceived health benefits, event awareness, event perception, education, income, occupation, gender, age, and residential area. The logit model estimates the effects of the proposed bid and respondent characteristics on the probability of accepting the bid. Maximum likelihood estimation was used to obtain the parameter estimates and identify the factors significantly associated with respondents' willingness to support the SIM as a public sport service.

#### Variables used in the model

The variables used to estimate the demand model in this study were derived from those previously validated in prior research (22, 39–42). Table 3 presents the independent variables used in the study, which are classified into four categories. First, variables related to respondents' usage patterns include participation frequency, interest in health, awareness and perception, membership in sports clubs, and the perceived necessity of public services (22, 39). Second, variables related to satisfaction after participation include satisfaction with the infrastructure (39). Third, variables related to the bid amount are included. Fourth, socio-demographic variables include age, gender, residential area, educational background, occupation, and monthly income (22, 41, 42). Bid amount, participation frequency, age, number of children, education level, and monthly income are measured on a ratio scale. Interest in health, interest in marathon participation, health benefits of marathon participation, and satisfaction with the infrastructure are measured on a 5-point Likert scale. All other variables are treated as dummy variables.

**Table 3:** Variable selection and classification.

Category	Variables
Dependent Variable	Willingness to pay
Independent Variables	Bid amount, interest in health, interest in marathon, satisfaction with infrastructure, club membership, event participation, health benefits from marathon, event awareness, event perception, education, income, occupation, gender, age, residential area

#### Estimation of non-market value

Among the 1,250 survey respondents, 829 (66.3%) indicated a positive willingness to pay (WTP), while 421 (33.7%) reported no WTP. For respondents who reported no WTP, a follow-up question was asked to clarify their reasons. Those who selected reasons such as “I do not have enough financial resources to pay,” “The project should be funded by taxes that I already pay,” “I do not trust that the local government will implement the plan as stated,” or “I doubt that the project will achieve the expected effects,” were treated as having a zero WTP related to objections to the payment vehicle or the allocation of public funds. Ultimately, the WTP estimation was based on data from 1,070 respondents, excluding protest responses, and included 829 respondents with positive WTP and 241 respondents with genuine zero WTP.

In this study, responses were classified as protest when respondents refused to pay due to objections to the tax-based payment vehicle or distrust in local government implementation, rather than because they perceived the SIM to have no value. Based on this criterion, 180 out of 1,250 responses were treated as protests and excluded from the WTP estimation, so the final analysis used 1,070 observations, including 829 positive WTP responses and 241 genuine zero WTP responses.

The non-market value estimated in this study using the Contingent Valuation Method (CVM) was Hicks' Compensation Variation (CV). The economic benefit corresponding to CV is measured by integrating the estimated function. First, the mean willingness to pay can be estimated by calculating the cumulative probability distribution from 0 to infinity (∞) (43). Second, the integration range can be set from the median bid amount to infinity (∞). Third, the truncated mean can be used by setting the integration range from 0 to the maximum bid amount and excluding the area beyond that point (44).

The choice of the integration range significantly influences the estimation of total economic value in CVM. This finding has been theoretically established by Hanemann (24) and subsequently confirmed in other studies. Comparative studies of various WTP statistics (e.g., mean, median, and truncated mean) and integration range settings, such as those by Borzykowski et al. (45) and Whitehead et al. (46), have repeatedly demonstrated that “integration from 0 to infinity (∞)” yields the most accurate estimates. Based on this evidence, this study applies and compares all three methods proposed by Hanemann (24).

## Results

### Results of the contingent valuation model estimation

In this study, the logit model, estimated using Maximum Likelihood Estimation (MLE), was employed as the demand model for non-market valuation. To verify the goodness-of-fit of the logit model, we examined the pseudo-R-squared statistics (Cox & Snell *R^2^,* Nagelkerke *R^2^*), the results of the Hosmer and Lemeshow test, and the predicted correct percentage (or predictive accuracy). The Hosmer and Lemeshow test indicated no significant difference between the predicted and observed values, and the predictive accuracy was 86.51%, suggesting that the model fit was adequate (Table 4).

**Table 4:** Results of CVM estimation (initial model).

Variable	B	S.E.	Wald	df	*p*-value	Exp(B)
Bid Amount	.000	.000	10.315	1	.001**	1.000
Interest in Health	-.041	.086	.225	1	.635	.960
Interest in Marathon	.365	.061	36.067	1	.000***	1.440
Satisfaction with Infrastructure	.594	.209	8.044	1	.005**	1.812
Club Membership	.153	.261	.344	1	.557	1.166
Event Participation	.018	.159	.013	1	.909	1.018
Marathon Health Benefit	-.317	.235	1.812	1	.178	.728
Event Awareness	-.173	.221	.613	1	.434	.841
Event Perception	.136	.057	5.769	1	.016**	1.145
Education	.040	.060	.435	1	.510	1.041
Income	-.043	.030	2.033	1	.154	.958
Occupation	.611	.169	13.075	1	.000***	1.842
Gender	-.292	.126	5.375	1	.020**	.746
Age	-.089	.053	2.739	1	.098	.915
Residential Area	.138	.137	1.016	1	.313	1.148
Constant	−1.284	1.311	.958	1	.328	.277
Log-likelihood	1,702.573					
Cox & Snell *R^2^*	.051					
Nagelkerke *R^2^*	.077					
Hosmer and Lemeshow	11.789					
Predicted correct	86.51%					

*p *<* *.*05, **p *<* *.*01, ***p *<* *.*001.

Based on Wald statistics, the model estimation results indicated that the bid amount, interest in marathon, satisfaction with infrastructure, event perception, occupation, and gender significantly influenced WTP (Table 5). In contrast, interest in health, club membership, event participation, health benefits from marathons, event awareness, education, income, age, and residential area did not significantly affect WTP and were thus excluded from the final model. Additionally, the bid amount was statistically significant at the 99% confidence level and had a negative effect on WTP. This indicates that as the bid amount increases, respondents' WTP decreases, and conversely, as the bid amount decreases, WTP increases.

**Table 5:** Results of the CVM estimation (final model).

Variable	B	S.E.	Wald	df	*p*-value	Exp(B)
Bid Amount	.000	.000	10.556	1	.001**	1.000
Interest in Marathon	.358	.059	36.382	1	.000***	1.431
Satisfaction with Infrastructure	.498	.177	7.924	1	.005**	1.646
Event Perception	.125	.053	5.639	1	.018*	1.134
Occupation	.591	.150	15.593	1	.000***	1.806
Gender	-.328	.124	6.945	1	.008**	.721
Constant	−2.647	.906	8.530	1	.003**	.071
Log-likelihood	1,710.477					
Cox & Snell *R^2^*	.046					
Nagelkerke *R^2^*	.070					
Hosmer and Lemeshow	19.950					
Predicted correct	78.61%					

*p *<* *.*05, **p *<* *.*01, ***p *<* *.*001.

Furthermore, variables with coefficient estimates significant at the 99.9% confidence level included interest in marathons and occupation, both of which had positive effects on WTP. This finding indicates that respondents with a greater interest in marathon events were more willing to pay, suggesting that individuals' psychological involvement and perceived value of marathon events may influence their support for investments in urban sport infrastructure. Variables significant at the 99% confidence level included satisfaction with infrastructure, event perception, and gender. Infrastructure satisfaction and event perception were found to positively (+) influence WTP, indicating that greater interest in the marathon and more positive perceptions of its infrastructure and the event lead to a higher WTP.

### Estimation results of the SIM's non-market value

In this study, the nonmarket value of the target project was estimated using all three welfare measures for WTP proposed by Hanemann (24). Variables that exhibited a statistically significant effect on willingness to pay were considered for the final model specification, and bid amount, interest in marathon events, satisfaction with related infrastructure, and perception of the event were retained as explanatory variables. Among these, only the bid amount was allowed to vary in the welfare calculations, while the remaining covariates were held at their sample means.

The estimated mean WTP was KRW 15,781.51, which exceeds the highest bid amount used in the survey. This result can be interpreted as indicating that the upper tail of the WTP distribution is overstated when information at the higher end of the distribution is limited in a double-bounded dichotomous choice framework. Previous work has noted that assuming log-normal or log-logistic distributions for WTP may further accentuate this pattern, leading to relatively large mean estimates compared with alternative specifications (47, 48).

In light of these considerations, the present study adopts a cautious approach to interpreting the mean WTP. Instead, it places primary emphasis on the truncated mean WTP, which attenuates the influence of extreme values and yields a more stable estimate. The truncated mean WTP obtained in this study was KRW 4,747.86, which is judged to reflect respondents' feasible payment capacity more realistically. The median WTP was KRW 14,394.95, providing a robust measure that is less sensitive to skewness and outliers. By reporting the mean, median, and truncated mean WTP together, the analysis provides a more comprehensive description of the underlying WTP distribution, with the truncated mean as the primary parameter for policy and practical interpretation. As shown in Table 6, based on bid amount, interest in marathon, satisfaction with infrastructure, and event perception, the per capita mean WTP was estimated at KRW 15,781.51 (USD 11.34), the median WTP at KRW 14,394.95 (USD 10.35), and the truncated WTP at KRW 4,747.86 (USD 3.41) (Table 6).

**Table 6:** Estimated non-market value of the SIM.

Category	WTP		
	Mean	Median	Truncated
WTP per person	15,781.51($11.34)	14,394.95($10.35)	4,747.86($3.41)

## Discussion

The present findings indicate that the SIM possesses measurable non-market value from the perspective of Seoul residents, as reflected in their willingness to pay. Rather than demonstrating the overall effectiveness of public policy or infrastructure investment, the estimated WTP provides an economic indicator of citizens' perceived value that may complement policy evaluation and resource allocation decisions. Because the estimated WTP captures both use and non-use values within the Total Economic Value framework, it reflects not only direct benefits from participation but also broader social values, such as civic pride and support for the event's continued existence. Accordingly, these findings should be interpreted as evidence of the perceived public value of the Seoul International Marathon rather than as definitive evidence of its overall economic or policy impact.

### Economic valuation of SIM using CVM

A review of previous studies on CVM shows that the survey design must be highly meticulous, and decisions regarding the survey population, WTP estimation methods, and payment vehicles must be made with great caution (14, 49). Steigenberger et al. (41), who systematically examined the health sector, suggested that the difference between actual and hypothetical WTP, survey environment, and the choice of elicitation method are major factors causing variation in results. Therefore, rigorous adherence to methodological procedures and careful consideration of key methodological variables are essential in CVM-based valuation, as they are crucial to enhancing the reliability of how results are interpreted and any policy application. This consistent finding in previous research highlights the importance of logical procedures and methodological caution when applying CVM. Accordingly, this study precisely selected a survey population aligned with the research objective and presented detailed, realistic scenarios, including the characteristics, scope, and expected effects of the event, to help respondents imagine real-world conditions. Furthermore, the double-bounded dichotomous choice method was adopted, and the starting point, range, and steps of the bid amount were logically and realistically determined to ensure the robustness of the results.

The empirical analysis revealed that bid amount, marathon interest, infrastructure satisfaction, event perception, occupation, and gender significantly affected respondents' WTP. The bid amount had a statistically significant negative effect at the 1% level, consistent with previous research findings indicating that respondents' actual WTP decreases as the bid amount increases (50, 51). Conversely, interest in marathons, satisfaction with infrastructure, and event perception had significant positive effects on WTP, indicating that respondents with more favorable perceptions of the event and related infrastructure placed a higher value on the non-market benefits. This finding is consistent with previous research demonstrating that individuals' emotional attachment, involvement, and perceived benefits associated with sport events positively influence their valuation of public sport services (51, 52). However, unlike studies that focus primarily on direct consumption benefits, such as event attendance or participation, the present study extends previous findings by demonstrating that psychological involvement with a mass-participation sporting event also contributes to the recognition of broader non-market values, including social cohesion and public health benefits. This suggests that the perceived value of large-scale sporting events is determined not only by individual participation benefits but also by the extent to which citizens view these events as socially meaningful public resources.

Furthermore, the per capita willingness-to-pay (WTP) estimated using Hanemann's (24) model was an average of 15,781 won (US$11.34), a median of 14,394 won (US$10.35), and a truncation value of 4,747 won (US$3.41). These findings reaffirm the usefulness of CVM as an effective tool for comprehensively capturing both use and non-use values for policy application. By calculating multiple WTP measures and averaging them, this study improves the accuracy of non-market value estimation, providing policymakers with more realistic information for informed decision-making.

### Determinants of willingness-to-pay

Analysis of the CVM results for the SIM revealed that the bid amount, number of participants, health interest, and perceived necessity significantly influenced WTP, as indicated by the Wald statistic. In particular, the number of participations, health interest, and perceived necessity had significant positive effects on demand, suggesting that respondents' positive perceptions and participation levels are key explanatory variables for evaluating use and non-use values. Both the event participation frequency and the number of visits to tourist sites showed a positive (+) effect on WTP, which aligns with findings from prior research (41, 52, 53). This result supports the argument that repeated exposure to sport events enhances familiarity, perceived benefits, and personal relevance, thereby increasing individuals' willingness to contribute financially. Similarly, higher health interest was associated with greater WTP, consistent with findings from previous studies. For example, McKenzie (54) demonstrated that individuals with greater interest in films exhibited higher WTP for film festivals, aligning with this study's findings.

Finally, respondents with a greater perceived necessity of the project showed higher WTP. This finding expands previous CVM research by highlighting perceived necessity as an important psychological mechanism underlying public support for sport-related investments. Previous studies have primarily examined the role of perceived necessity in areas such as environmental sustainability, transportation, and healthcare (55, 56), whereas its application to sport policy evaluation has received limited attention. Consistent with previous findings, which demonstrated that stronger perceptions of necessity positively influence WTP and purchase intention, the present study shows that citizens who perceive a marathon event as necessary for achieving broader social objectives, including health promotion, urban vitality, and community integration, are more likely to assign greater economic value to the event. However, unlike previous studies focusing on individual consumption decisions or service adoption, this study extends the concept of perceived necessity to the valuation of a public sport service, demonstrating its role in shaping public acceptance and policy legitimacy. Therefore, perceived necessity should be considered a key factor in designing and evaluating urban sport infrastructure policies.

The SIM is an internationally prestigious event, having received the World Athletics Heritage Plaque in May 2095. Together with the Boston Marathon, it is one of only two events worldwide to hold both the Platinum Label and Heritage Plaque certifications. According to the 2025 Brand Finance report, the SIM ranked 37th among the world's top 50 marathons in terms of economic impact, generating an estimated USD 27 million in economic benefits for the host city (57). This outcome is attributed to runners' strong commitment to well-being, the growing popularity of running, its domestic and international appeal, an innovative spirit that embraces new technologies, and dedication to sustainability, all of which have contributed to significant economic spillover effects.

### Strategic implications and policy applications

The economic valuation results from this study provide not only an estimate of the current public value of the Seoul International Marathon (SIM) but also strategic guidance for its future development. Recent best practices among leading international marathons indicate a shift from elite performance-centered events toward more inclusive, socially cohesive, and environmentally responsible formats that foster community solidarity and local development (58, 59). In this context, the SIM should evolve from a performance-oriented sporting event into a public sports platform that encourages participation by people with disabilities, older adults, and youth, thereby strengthening its role as a civic program for health promotion and social inclusion. Recent best practices among leading international marathons indicate a shift from elite performance-centered events toward more inclusive, socially cohesive, and environmentally responsible formats that foster community solidarity and local development (58, 59). This strategic direction is consistent with the determinants of willingness to pay identified in this study, particularly perceived necessity, health interest, and participation frequency. The positive association between residents' perceptions of the event and their willingness to pay may reflect broader sociocultural benefits reported in previous studies. Major sporting events have been shown to strengthen community identity, civic pride, and social cohesion, all of which contribute to public support for continued investment in such events (6, 60). Accordingly, enhancing the SIM's inclusiveness and social relevance is likely to increase citizens' perceived necessity for the event and, in turn, its non-market value.

More broadly, the findings show that sports events as public services can generate meaningful economic value through the systematic measurement of use and non-use values. The estimated willingness-to-pay figures capture not only direct participation benefits but also intangible social benefits such as community solidarity, health promotion, and social integration, which are often overlooked in conventional economic analyses. This comprehensive valuation approach provides policymakers with quantified evidence to support decisions on event sustainability, resource allocation, and policy prioritization. In addition, the non-market valuation framework provides a baseline for future policy evaluation by allowing subsequent interventions to be assessed against changes in perceived public value.

Ultimately, this study shows that rigorous measurement of non-market value through contingent valuation can make a meaningful contribution to rational, evidence-based decision-making in public sports management and urban governance. The methodological approach and empirical findings presented here may also be extended to the evaluation of other public events and services, thereby supporting broader evidence-based policymaking in the public sector.

### Practical implications

The findings of this study yield several practical implications for policymakers and event organizers. First, the bid amount emerged as a significant determinant of willingness to pay, indicating that citizens' financial support is sensitive to the proposed contribution level. This suggests that public funding mechanisms for the SIM should be designed with careful attention to affordability and perceived acceptability. Second, the positive willingness to pay observed among respondents provides empirical support for maintaining or moderately expanding public investment in the event as a form of urban sport infrastructure and public sport service. Third, perceptions of the marathon's public value significantly increased willingness to pay, indicating that policy communication should emphasize the event's broader contributions to public health, tourism, urban branding, and social cohesion. More broadly, the estimated willingness-to-pay structure and its determinants may be incorporated into municipal sport and health planning as evidence to prioritize investment, improve supporting infrastructure, and align event strategy with wider urban policy objectives. In this respect, the present study extends previous non-market valuation research by demonstrating the policy relevance of citizen-based valuation for urban marathons.

### Limitations and future research

This study has several limitations that should be considered when interpreting the findings. First, as with all contingent valuation method (CVM) studies, the estimated willingness to pay (WTP) was based on hypothetical scenarios rather than actual market behavior, which may have resulted in hypothetical bias. Although bid amounts were determined through a pilot survey, the maximum bid of KRW 6,000 may not have fully captured the preferences of respondents with relatively high WTP. In addition, protest responses were excluded from the main analysis because they reflected objections to the payment vehicle or distrust in government implementation rather than genuine zero valuations. While this approach is consistent with common CVM practice, it may have led to a modest upward bias in the estimated mean WTP. Second, the sample consisted exclusively of Seoul residents. Although demographic quotas were applied, the findings reflect the preferences of a geographically specific population with relatively high familiarity with the Seoul International Marathon, thereby limiting their generalizability to the broader Korean population.

Future research should address these limitations by employing broader bid ranges or alternative elicitation formats, comparing different approaches to handling protest responses, and using nationally representative samples to enhance the generalizability of the findings. In addition, advanced statistical techniques, such as Multiple Imputation (MI), should be considered to account for protest responses and to assess the robustness of WTP estimates under alternative data-treatment approaches. In addition, future studies should integrate environmental sustainability attributes into economic valuation models and complement quantitative findings with qualitative evidence from policymakers and event participants to develop more comprehensive and actionable policy recommendations.

## Conclusion

The SIM transcends the realm of a simple private sports event, serving as a public service that generates multifaceted social value, including large-scale civic participation, public health promotion, collaboration with urban governance, enhancement of the global city image, tourism revitalization, and intergenerational and cross-class integration. This study evaluated the public service role of the SIM by applying the CVM to quantitatively estimate its potential economic value, providing an empirical foundation for policy implementation. This study also identified factors influencing citizens' willingness to pay (WTP), providing further evidence of the SIM's public value. The findings indicate that citizens expressed a positive willingness to financially support the event, recognizing its diverse social and public benefits. In particular, bid amount, event awareness, and perceptions of the marathon's public value emerged as significant determinants of WTP. These findings suggest that citizens' willingness to support the event is influenced not only by economic considerations but also by their recognition of its broader social contributions.

Recently, studies evaluating the economic value of urban sports events, such as the SIM, have garnered attention as exemplary cases of empirical research, both in terms of academic significance and policy relevance. In particular, CVM is established as a representative non-market valuation method for objectively assessing the socio-economic impact of large-scale public sports events using scientific evidence, thereby providing empirical data for effective policy-making and event management. Moreover, this study extends the existing literature by providing empirical evidence from one of Asia's largest urban marathons. It demonstrates the applicability of the CVM as a policy evaluation tool for public sport services.

## Data Availability

The raw data supporting the conclusions of this article will be made available by the authors, without undue reservation.

## Supplementary material

SUPPLEMENTARY APPENDIX A

Pilot questionnaire.

SUPPLEMENTARY APPENDIX B

Perception Survey of Economic Valuation of the Seoul International Marathon as Urban Sport Infrastructure (Version A).

SUPPLEMENTARY APPENDIX C

Perception Survey of Economic Valuation of the Seoul International Marathon as Urban Sport Infrastructure (Version B).
