# Supplementary material for: Valuing an urban marathon as a public sport service: evidence from the Seoul international marathon
Source: Front Sports Act Living. 2026 Sep 30;8:1896016. doi: 10.3389/fspor.2026.1896016 (PMC13661926; doi:10.3389/fspor.2026.1896016)
Supplement: Supplementary file 1 [file Supplementaryfile1.pdf]

Appendix A. Pilot questionnaire

Article 33 of the Statistics Act (Protection of Confidentiality, etc.)<br>Matters learned in the process of compiling statistics that pertain to the secrets of an individual, corporation, or organization shall be protected.	ID				

Hello.

This survey is conducted under the auspices of the Seoul Sports Council as a "questionnaire on citizens' perceptions related to the Seoul International Marathon." The survey targets Seoul citizens and aims to collect their overall perceptions of the Seoul International Marathon to be used as basic data for formulating sports policies.

This questionnaire has been prepared and approved under the supervision of the Ministry of Culture, Sports and Tourism. In accordance with Article 33 of the Statistics Act (revised in December 1996), your responses will never be used for purposes other than research, and all personal information will be kept strictly confidential. Participation in this study is entirely voluntary, and there will be no disadvantages or penalties for choosing not to participate. Participants may withdraw from or discontinue their participation at any time without having to provide any reason, and they will not incur any disadvantage as a result of such withdrawal. There are no right or wrong answers in this survey. For the accuracy of the study, we kindly ask you to take a moment to respond honestly.

I have read and understood all of the above information, and I voluntarily agree to participate in this study. Agree □ / Do not agree □

1 June 2025

Principal Investigator: Jae-Eun Shin

Affiliation: Chung-Ang University

Contact: 010-4233-8820

Part I. Basic information about the respondent

1. Residential Area

① Jongno-gu ②Jung-gu ③Yongsan-gu ④Seongdong-gu ⑤Gwangjin-gu

⑥Dongdaemun-gu ⑦Jungnang-gu ⑧Seongbuk-gu ⑨Gangbuk-gu ⑩Dobong-gu

⑪ Nowon-gu ⑫ Eunpyeong-gu ⑬ Seodaemun-gu ⑭ Mapo-gu ⑮Yangcheon-gu

⑯ Gangseo-gu ⑰ Guro-gu ⑱ Geumcheon-gu ⑲ Dongjak-gu ⑳ Yeongdeungpo-gu

㉑ Gwanak-gu ㉒ Seocho-gu ㉓ Gangnam-gu ㉔ Songpa-gu ㉕ Gangdong-gu

2. Gender

① Male ② Female

3. Age

Age in full years: _______ years

① 20s ② 30s ③ 40s ④ 50s ⑤ 60s

Part II. Questions on the intangible benefits of the Seoul International Marathon

※ This survey is conducted to estimate the social value of the Seoul International Marathon, which has been selected by World Athletics as one of the world's three great marathon heritage events.

In Seoul, around 100 marathon events of various types are held annually. Among them, the Seoul International Marathon hosted by the Seoul Metropolitan Government is a Gold Label race, the highest grade recognized by World Athletics. In addition, various international marathon events such as the Seoul International Wheelchair Marathon are also held.

Q1. Were you aware that an international marathon event is held in Seoul every year?

① I knew about it accurately.

② I did not know the exact name, but I knew that there is such an event.

③ I did not know at all.

Q2. What is your overall opinion of the Seoul International Marathon held in Seoul?

①	②	③	④	⑤

Very negative　　Negative　　Neutral　　Positive　　Very positive

Q3. What do you think is the most positive aspect of the Seoul International Marathon? (Please choose two.)

① It enhances the image of Seoul and contributes to the city's competitiveness.

② It can contribute to the development of the local industrial economy and tourism.

③ It contributes to activating sports infrastructure and expanding the base of sport for all in Seoul.

④ It enhances citizens' pride and serves as an opportunity for socio-cultural advancement.

⑤ Other (　　　　　　)

Q4. What do you think is the most negative aspect of the Seoul International Marathon? (Please choose two.)

① It will lead to economic losses, such as tax expenditures associated with hosting the event.

② Public opinion is not sufficiently collected, and the event will be used for the political purposes of a few, such as local government heads.

③ The effects gained from holding the event will not be significant.

④ There will be many inconveniences, such as control of urban roads for the event.

⑤ Other (　　　　　　)

Q5. In your opinion, in which direction should the Seoul International Marathon be developed?

① Course design that can promote Seoul internationally.

② Maximizing tourism effects through the event.

③ Enhancing the status of the event by inviting elite athletes.

④ Turning the event into a festival through the empathy and participation of Seoul citizens.

⑤ Other (　　　　　　)

Part III. Questions on the intangible benefits of the Seoul International Marathon

※ This survey is conducted to estimate the economic value of the Seoul International Marathon, which has been selected by World Athletics as one of the world's three great marathon heritage events.

The following statements describe potential benefits that may arise from hosting the Seoul International Marathon. First, as the capital of the Republic of Korea, Seoul can enhance its brand

image and promote the national image, which in turn is expected to increase domestic companies' sales. In addition, during the event period, domestic and international leisure, sports, and tourism activities are invigorated, thereby contributing to the growth of related industries. The event also has various effects such as boosting the pride and morale of Seoul citizens.

Suppose that the Seoul Metropolitan Government can raise additional financial resources to enhance the value of the Seoul International Marathon by creating a fund through voluntary donations from citizens. Considering both the benefits and costs, please answer the following questions.

Q1. If a fund were created through donations for the successful hosting of the Seoul International Marathon, would you be willing to contribute?

① Yes ( ) → Go to Q2

② No ( ) → Go to Q3

Q2. How much would you be willing to donate to the fund for the successful hosting of the Seoul International Marathon?

Amount you are willing to pay: ________ KRW

Q3. What is the most important reason you would not be willing to pay (e.g., in the form of additional income tax) for the successful hosting of the Seoul International Marathon?

① I do not have sufficient economic resources to pay.

② It should be financed by taxes that have already been collected.

③ I do not trust that the local government will implement the plan as stated.

④ The Seoul International Marathon is not of interest to me.

⑤ I doubt that the expected effects will be realized.

⑥ I have not been given sufficient information to make a judgment.

⑦ Other ( )

Q4. What is the most important reason you would be willing to pay (e.g., in the form of additional income tax) for the successful hosting of the Seoul International Marathon?

① As a citizen of Seoul, to increase my pride in the event.

② To strengthen city promotion through the successful hosting of the event.

③ To promote the activation of the domestic economy through related industries and tourism in Seoul.

④ To promote the activation of marathon as a sport for all.

⑤ Because I trust the policies of the local government.

⑥ Other (   )

Part V. Demographic questions

Q1. What is your highest level of education?

① No formal schooling ② Elementary school graduate ③ Middle school graduate

④ High school graduate ⑤ Currently enrolled in university ⑥ University graduate

⑦ Currently enrolled in graduate school ⑧ Graduate school graduate

Q2. What is the approximate total monthly household income of all members combined?

① Less than 1 million KRW ② 1 million – less than 2 million KRW

③ 2 million – less than 3 million KRW ④ 3 million – less than 4 million KRW

⑤ 4 million – less than 5 million KRW ⑥ 5 million – less than 6 million KRW

⑦ 6 million – less than 7 million KRW ⑧ 7 million – less than 8 million KRW

⑨ 8 million – less than 9 million KRW ⑩ 9 million – less than 10 million KRW

⑪ 10 million KRW or more (________ KRW)

Q3. Do you currently have a full-time occupation?

① Unemployed ② Office/administrative worker ③ Homemaker ④ University student

⑤ Self-employed ⑥ Professional ⑦ Research/technical worker ⑧ Sales/service worker

⑨ Production/technical worker ⑩ Public servant (including teachers)

⑪ Agriculture, livestock, forestry, or fishery ⑫ Other (   )

Appendix B. Perception Survey of Economic Valuation of the Seoul International Marathon as Urban Sport Infrastructure (Version A)

Article 33 of the Statistics Act (Protection of Confidentiality, etc.)<br>Matters learned in the process of compiling statistics that pertain to the secrets of an individual, corporation, or organization shall be protected.	ID | | | |

Hello.

This survey is conducted under the auspices of the Seoul Sports Council as a "questionnaire on citizens' perceptions related to the Seoul International Marathon." The survey targets Seoul citizens and aims to collect their overall perceptions of the Seoul International Marathon to be used as basic data for formulating sports policies.

This questionnaire has been prepared and approved under the supervision of the Ministry of Culture, Sports and Tourism. In accordance with Article 33 of the Statistics Act (revised in December 1996), your responses will never be used for purposes other than research, and all personal information will be kept strictly confidential. Participants may withdraw from or discontinue their participation at any time without having to provide any reason, and they will not incur any disadvantage as a result of such withdrawal. There are no right or wrong answers in this survey. For the accuracy of the study, we kindly ask you to take a moment to respond honestly.

I have read and understood all of the above information, and I voluntarily agree to participate in this study. Agree □ / Do not agree □

1 June 2025

Principal Investigator: Jae-Eun Shin

Affiliation: Chung-Ang University

Contact: 010-4233-8820

Part I. Basic information about the respondent

1. Residential area

①Jongno-gu ②Jung-gu ③Yongsan-gu ④Seongdong-gu ⑤Gwangjin-gu

⑥Dongdaemun-gu ⑦Jungnang-gu ⑧Seongbuk-gu ⑨Gangbuk-gu ⑩Dobong-gu

⑪Nowon-gu ⑫Eunpyeong-gu ⑬Seodaemun-gu ⑭Mapo-gu ⑮Yangcheon-gu

⑯ Gangseo-gu ⑰ Guro-gu ⑱ Geumcheon-gu ⑲ Dongjak-gu

⑳ Yeongdeungpo-gu ㉑Gwanak-gu ㉒ Seocho-gu ㉓Gangnam-gu

㉔Songpa-gu ㉕Gangdong-gu

2. Gender

① Male ② Female

3. Age

Age in full years: ______ years

① 20s ② 30s ③ 40s ④ 50s ⑤ 60s

Part I-A. Questions on your health and exercise tendencies

Q1. How interested are you in your health in everyday life?

①Not interested at all ②Not interested ③Average ④Interested ⑤Very interested

Q2. How interested are you in running or marathon in everyday life?

①Not interested at all ②Not interested ③Average ④Interested ⑤Very interested

Q3. What type of running exercise do you usually do for health?

① Walking ② Jogging ③ Club activities ④ Other ( )

Q4. In your opinion, what is needed to improve the running environment in Seoul?

① Development of various walking and running courses

② Expansion of free training classes

③ Support for hosting various events and races

④ Provision of various running-related information

⑤ Other ( )

Q5. Overall, how do you feel about the running infrastructure in Seoul?

① Very dissatisfied ② Dissatisfied ③ Average ④ Satisfied ⑤ Very satisfied

Q6. Are you currently participating in a marathon or other sports club?

① Marathon club ② Other sports club ③ No

Q7. Do you have experience participating in marathon or other sports events?

① Marathon event(s) ② Other sports events ③ No

Q8. To what extent has running or marathon been helpful to your health?

① Very dissatisfied ② Dissatisfied ③ Average ④ Satisfied ⑤ Very satisfied

Q9. Would you be willing to recommend running or marathon to people around you?

① No willingness to recommend at all

② Not willing to recommend

③ Average

④ Willing to recommend

⑤ Very willing to recommend

Part II. Questions on the intangible benefits of the Seoul International Marathon

※ This survey is conducted to estimate the social value of the Seoul International Marathon, which has been selected by World Athletics as one of the world's three great marathon heritage events.

> In Seoul, around 100 marathon events of various types are held annually. Among them, the Seoul International Marathon hosted by the Seoul Metropolitan Government is a Gold Label race, the highest grade recognized by World Athletics. In addition, various international marathon events such as the Seoul International Wheelchair Marathon are also held.

Q1. Were you aware that an international marathon event is held in Seoul every year?

① I knew about it accurately.

② I did not know the exact name, but I knew that there is such an event.

③ I did not know at all.

Q2. What is your overall opinion of the Seoul International Marathon held in Seoul?

① Very negative ② Negative ③ Neutral ④ Positive ⑤ Very positive

Q3. What do you think is the most positive aspect of the Seoul International Marathon? (Please choose two.)

① It enhances the image of Seoul and contributes to the city's competitiveness.

② It can contribute to the development of the local industrial economy and tourism.

③ It contributes to activating sports infrastructure and expanding the base of sport for all in Seoul.

④ It enhances citizens' pride and serves as an opportunity for socio-cultural advancement.

⑤ Other (   )

Q4. What do you think is the most negative aspect of the Seoul International Marathon? (Please choose two.)

① It will lead to economic losses, such as tax expenditures associated with hosting the event.

② Public opinion is not sufficiently collected, and the event will be used for the political purposes of a few, such as local government heads.

③ The effects gained from holding the event will not be significant.

④ There will be many inconveniences, such as control of urban roads for the event.

⑤ Other (   )

Q5. In your opinion, in which direction should the Seoul International Marathon be developed?

① Designing marathon courses that enhance Seoul's international image.

② Maximizing tourism effects through the event.

③ Enhancing the status of the event by inviting elite athletes.

④ Turning the event into a festival through the empathy and participation of Seoul citizens.

⑤ Other (   )

Part III. Questions on the intangible benefits of the Seoul International Marathon

※ This survey is conducted to estimate the economic value of the Seoul International Marathon, which has been selected by World Athletics as one of the world's three great marathon heritage events.

The following statements describe potential benefits that may arise from hosting the Seoul International Marathon. First, as the capital of the Republic of Korea, Seoul can enhance its brand image and promote the national image, which in turn is expected to increase domestic companies' sales. In addition, during the event period, domestic and international leisure, sports, and tourism activities are invigorated, thereby contributing to the growth of related industries. The event also has various effects such as boosting the pride and morale of Seoul citizens.

Suppose that the Seoul Metropolitan Government can raise additional financial resources to enhance the value of the Seoul International Marathon by creating a fund through voluntary donations from citizens. Considering both the benefits and costs, please answer the following questions.

Q1. If a fund were created through donations for the successful hosting of the Seoul International Marathon, would you be willing to contribute? (Donations are used to improve competition quality, facilities, environmental sustainability, safety, and participant services.)

① Yes ( ) → Go to Q2 ② No ( ) → Go to Q5

Q2. Would you be willing to pay a donation of 2,000 KRW into such a fund for the successful hosting of the Seoul International Marathon?

① Yes ( ) → Go to Q3 ② No ( ) → Go to Q4

Q3. (For those who answered ① in Q2 only) You stated that you are willing to pay a donation of 2,000 KRW; would you also be willing to pay 3,000 KRW as a donation into the fund?

① Yes ( ) → Go to Q6 ② No ( ) → Go to Q6

Q4. (For those who answered ② in Q1 only) In that case, would you be willing to pay a donation of 1,000 KRW into the fund?

① Yes ( ) → Go to Q6 ② No ( ) → Go to Q5

Q5. What is the most important reason you would not be willing to pay (e.g., in the form of additional income tax) for the successful hosting of the Seoul International Marathon?

① I do not have sufficient economic resources to pay.

② It should be financed by taxes that have already been collected.

③ I do not trust that the local government will implement the proposed funding plan as stated.

④ The Seoul International Marathon is not of interest to me.

⑤ I doubt that the expected effects will be realized.

⑥ I have not been given sufficient information to make a judgment.

⑦ Other ( )

Q6. What is the most important reason you would be willing to pay (e.g., in the form of additional income tax) for the successful hosting of the Seoul International Marathon?

① As a citizen of Seoul, to increase my pride in the event.

② To strengthen city promotion through the successful hosting of the event.

③ To promote the activation of the domestic economy through related industries and tourism in Seoul.

④ To promote the activation of marathon as a sport for all.

⑤ Because I trust the policies of the local government.

⑥ Other (   )

Part V. Demographic questions

Q1. What is your highest level of education?

① No formal schooling ② Elementary school graduate ③ Middle school graduate

④ High school graduate ⑤ Currently enrolled in university ⑥ University graduate

⑦ Currently enrolled in graduate schoo l⑧ Graduate school graduate

Q2. What is the approximate total monthly household income of all members combined?

① Less than 1 million KRW ② 1 million – less than 2 million KRW

③ 2 million – less than 3 million KRW ④ 3 million – less than 4 million KRW

⑤ 4 million – less than 5 million KRW ⑥ 5 million – less than 6 million KRW

⑦ 6 million – less than 7 million KRW ⑧ 7 million – less than 8 million KRW

⑨ 8 million – less than 9 million KRW ⑩ 9 million – less than 10 million KRW

⑪ 10 million KRW or more (_________ KRW)

Q3. Do you currently have a full-time occupation?

① Unemployed ② Office/administrative worker ③ Homemaker ④ University student

⑤ Self-employed ⑥ Professional ⑦ Research/technical worker

⑧ Sales/service worker ⑨ Production/technical worker ⑩ Public servant (including teachers) ⑪ Agriculture, livestock, forestry, or fishery ⑫ Other (   )

Appendix C. Perception Survey of Economic Valuation of the Seoul International Marathon as Urban Sport Infrastructure (Version B)

Article 33 of the Statistics Act (Protection of Confidentiality, etc.)<br>Matters learned in the process of compiling statistics that pertain to the secrets of an individual, corporation, or organization shall be protected.	ID				

Hello.

This survey is conducted under the auspices of the Seoul Sports Council as a "questionnaire on citizens' perceptions related to the Seoul International Marathon." The survey targets Seoul citizens and aims to collect their overall perceptions of the Seoul International Marathon to be used as basic data for formulating sports policies.

This questionnaire has been prepared and approved under the supervision of the Ministry of Culture, Sports and Tourism. In accordance with Article 33 of the Statistics Act (revised in December 1996), your responses will never be used for purposes other than research, and all personal information will be kept strictly confidential. Participants may withdraw from or discontinue their participation at any time without having to provide any reason, and they will not incur any disadvantage as a result of such withdrawal. There are no right or wrong answers in this survey. For the accuracy of the study, we kindly ask you to take a moment to respond honestly.

I have read and understood all of the above information, and I voluntarily agree to participate in this study. Agree □ / Do not agree □

1 June 2025

Principal Investigator: Jae-Eun Shin

Affiliation: Chung-Ang University

Contact: 010-4233-8820

Part I. Basic information about the respondent

1. Residential area

①Jongno-gu ②Jung-gu ③Yongsan-gu ④Seongdong-gu ⑤Gwangjin-gu

⑥Dongdaemun-gu ⑦Jungnang-gu ⑧Seongbuk-gu ⑨Gangbuk-g ⑩Dobong-gu

⑪Nowon-gu ⑫Eunpyeong-gu ⑬Seodaemun-gu ⑭Mapo-gu ⑮Yangcheon-gu

⑯ Gangseo-gu ⑰ Guro-gu ⑱ Geumcheon-gu ⑲Dongjak-gu ⑳ Yeongdeungpo-gu

㉑ Gwanak-gu ㉒ Seocho-gu ㉓Gangnam-gu ㉔ Songpa-gu ㉕ Gangdong-gu

2. Gender

① Male ② Female

3. Age

Age in full years: _______ years

① 20s ② 30s ③ 40s ④ 50s ⑤ 60s

Part II. Questions on the intangible benefits of the Seoul International Marathon

※ This survey is conducted to estimate the social value of the Seoul International Marathon, which has been selected by World Athletics as one of the world's three great marathon heritage events.

In Seoul, around 100 marathon events of various types are held annually. Among them, the Seoul International Marathon hosted by the Seoul Metropolitan Government is a Gold Label race, the highest grade recognized by World Athletics. In addition, various international marathon events such as the Seoul International Wheelchair Marathon are also held.

Q1. Were you aware that an international marathon event is held in Seoul every year?

① I knew about it accurately.

② I did not know the exact name, but I knew that there is such an event.

③ I did not know at all.

Q2. What is your overall opinion of the Seoul International Marathon held in Seoul?

① Very negative ② Negative ③ Neutral ④ Positive ⑤ Very positive

Q3. What do you think is the most positive aspect of the Seoul International Marathon? (Please choose two.)

① It enhances the image of Seoul and contributes to the city's competitiveness.

② It can contribute to the development of the local industrial economy and tourism.

③ It contributes to activating sports infrastructure and expanding the base of sport for all in Seoul.

④ It enhances citizens' pride and serves as an opportunity for socio-cultural advancement.

⑤ Other (   )

Q4. What do you think is the most negative aspect of the Seoul International Marathon? (Please choose two.)

① It will lead to economic losses, such as tax expenditures associated with hosting the event.

② Public opinion is not sufficiently collected, and the event will be used for the political purposes of a few, such as local government heads.

③ The effects gained from holding the event will not be significant.

④ There will be many inconveniences, such as control of urban roads for the event.

⑤ Other (   )

Q5. In your opinion, in which direction should the Seoul International Marathon be developed?

① Designing marathon courses that enhance Seoul's international image.

② Maximizing tourism effects through the event.

③ Enhancing the status of the event by inviting elite athletes.

④ Turning the event into a festival through the empathy and participation of Seoul citizens.

⑤ Other (   )

Part III. Questions on the intangible benefits of the Seoul International Marathon

※ This survey is conducted to estimate the economic value of the Seoul International Marathon, which has been selected by World Athletics as one of the world's three great marathon heritage events.

The following statements describe potential benefits that may arise from hosting the Seoul International Marathon. First, as the capital of the Republic of Korea, Seoul can enhance its brand image and promote the national image, which in turn is expected to increase domestic companies' sales. In addition, during the event period, domestic and international leisure, sports, and tourism activities are invigorated, thereby contributing to the growth of related industries.

The event also has various effects such as boosting the pride and morale of Seoul citizens.<br><br>Suppose that the Seoul Metropolitan Government can raise additional financial resources to enhance the value of the Seoul International Marathon by creating a fund through voluntary donations from citizens. Considering both the benefits and costs, please answer the following questions.

Q1. If a fund were created through donations for the successful hosting of the Seoul International Marathon, would you be willing to contribute? (Donations are used to improve competition quality, facilities, environmental sustainability, safety, and participant services.)

① Yes ( ) → Go to Q2 ② No ( ) → Go to Q5

Q2. Would you be willing to pay a donation of 5,000 KRW into such a fund for the successful hosting of the Seoul International Marathon?

① Yes ( ) → Go to Q3 ② No ( ) → Go to Q4

Q3. (For those who answered ① in Q2 only) You stated that you are willing to pay a donation of 2,000 KRW; would you also be willing to pay 5,000 KRW as a donation into the fund?

① Yes ( ) → Go to Q6 ② No ( ) → Go to Q6

Q4. (For those who answered ② in Q1 only) In that case, would you be willing to pay a donation of 4,000 KRW into the fund?

① Yes ( ) → Go to Q6 ② No ( ) → Go to Q5

Q5. What is the most important reason you would not be willing to pay (e.g., in the form of additional income tax) for the successful hosting of the Seoul International Marathon?

① I do not have sufficient economic resources to pay.

② It should be financed by taxes that have already been collected.

③ I do not trust that the local government will implement the proposed funding plan as stated.

④ The Seoul International Marathon is not of interest to me.

⑤ I doubt that the expected effects will be realized.

⑥ I have not been given sufficient information to make a judgment.

⑦ Other ( )

Q6. What is the most important reason you would be willing to pay (e.g., in the form of

additional income tax) for the successful hosting of the Seoul International Marathon?

① As a citizen of Seoul, to increase my pride in the event.

② To strengthen city promotion through the successful hosting of the event.

③ To promote the activation of the domestic economy through related industries and tourism in Seoul.

④ To promote the activation of marathon as a sport for all.

⑤ Because I trust the policies of the local government.

⑥ Other (   )

Part V. Demographic questions

Q1. What is your highest level of education?

① No formal schooling ② Elementary school graduate ③ Middle school graduate

④ High school graduate ⑤ Currently enrolled in university ⑥ University graduate

⑦ Currently enrolled in graduate school ⑧ Graduate school graduate

Q2. What is the approximate total monthly household income of all members combined?

① Less than 1 million KRW ② 1 million – less than 2 million KRW

③ 2 million – less than 3 million KRW ④ 3 million – less than 4 million KRW

⑤ 4 million – less than 5 million KRW ⑥ 5 million – less than 6 million KRW

⑦ 6 million – less than 7 million KRW ⑧ 7 million – less than 8 million KRW

⑨ 8 million – less than 9 million KRW ⑩ 9 million – less than 10 million KRW

⑪ 10 million KRW or more (________ KRW)

Q3. Do you currently have a full-time occupation?

① Unemployed ② Office/administrative worker ③ Homemaker ④ University student

⑤ Self-employed ⑥ Professional ⑦ Research/technical worker ⑧ Sales/service worker

⑨ Production/technical worker ⑩ Public servant (including teachers)

⑪ Agriculture, livestock, forestry, or fishery ⑫ Other (   )
